# Plants for human health: greening biotechnology and synthetic biology

**DOI:** 10.1093/jxb/erx268

**Published:** 2017-09-04

**Authors:** Tessa Moses, Alain Goossens

**Affiliations:** Ghent University, Department of Plant Biotechnology and Bioinformatics, Ghent, Belgium; VIB Center for Plant Systems Biology, Ghent, Belgium

**Keywords:** Functional foods, flavonoids, natural products, next-generation sequencing, phytochemicals, synthetic biology, transporters, Functional foods, flavonoids, natural products, next-generation sequencing, phytochemicals, synthetic biology, transporters

**The concept of growing plants for human health and general well-being, rather than for consumption as food alone, is changing people’s perception of plant biotechnology and synthetic biology. Resurrecting the long-forgotten connection between plants and health has launched a new generation of botanical therapeutics, which include dietary supplements, functional foods, pharmaceuticals and multi-component drug mixtures. Technological and methodological advances have made the discovery, validation and manufacture of high-value phytochemicals a reality, and botanical therapeutics have come of age.**

Plants are integral to human well-being. In addition to their obvious nutritional value, for centuries they have also been used for their healing properties. Plant parts, potions and powders have been, and continue to be, used in traditional medicine by a number of tribes with varying degrees of success to boost vigor, as well as to prevent and cure disease. Archaeological evidence suggests the use of plants by humans for medicinal purposes during the Paleolithic age, with the first written evidence dating back to the Sumerians (Sumner, 2000).

By the 21st century, the pharmaceutical industry had mostly replaced natural plant extracts with chemically synthesized molecules to prevent and treat diseases. With the introduction of medicine in the form of easy to consume ‘pills’, it is easy to dissociate plants from health and thus overlook the many modern medicines which still contain phytochemicals in their natural form or as derivatives. Until the 20th century plant extracts were regularly screened for novel pharmaceutically active compounds, which were then purified from the native plants. The emphasis of the pharmaceutical industry then shifted to making these natural products synthetically, and using them as templates for generating structural analogues as a means of obtaining new chemical entities with desired efficacy.

The richness and diversity of novel drugs that can be discovered from plants have also been challenged by competition from the fields of combinatorial chemistry (Pandeya and Thakkar, 2005; Liu *et al.*, 2017) and computational drug design (Sliwoski *et al.*, 2014). It can be argued that several years of bioprospecting have resulted in the identification of the most relevant and relatively abundant plant natural compounds with pharmaceutical value. However, it is possible that several high-value plant-derived compounds with pharmacological activity still remain undiscovered, either because they are produced in plants which are not easily accessible or due to a lack of advanced methodologies (Mendelsohn and Balick, 1995; Van Wyk, 2015). All living plant species in the world together contribute to a greater chemical diversity of bioactive compounds than any man-made chemical library, and therefore finding novel plant molecules today would require more sophisticated discovery approaches.

## Engineered well-ness

In addition to the pharmacological relevance of plant natural products, there is a growing trend of boosting human well-ness by incorporating plant parts and products into daily diets in the form of functional foods and dietary supplements (Box 1). The regular consumption of certain fruits and vegetables is suggested to reduce the risk of chronic diseases like cancer, diabetes, heart diseases and obesity by functioning as medicinal nutrition therapy (Martin *et al.*, 2013). Flavonoids comprise one such class of phytochemicals that is commonly found in fruits, vegetables and some beverages including wine. Flavonoids are polyphenolic compounds with health-promoting biochemical and antioxidant effects that are beneficial for nutraceutical, pharmaceutical and cosmetic applications. This widely distributed class of plant specialized metabolites comprises over 8000 compounds bearing a common diphenylpropane backbone; these are found in several parts of the plant and are commonly recognized as flower pigments (Panche *et al.*, 2016). It is estimated that the Western population consumes 20–50 mg of these compounds in their diet daily. Tohge *et al.* (2017) present an overview of the chemical diversity of flavonoids across plant species, and showcase the impact of technical advancements in making better phytochemical inventories for plants, with a focus on the model plant Arabidopsis and the crop plants tomato, maize, rice and beans. The increased accessibility of whole-genome sequencing, together with reverse genetics and molecular phenotyping, have greatly contributed to the current understanding of flavonoid metabolic networks (Tohge *et al.*, 2017). The data emerging from the 1001 Arabidopsis genomes project and the 1000 plants project will enrich the structure-to-function relationships of flavonoids to benefit translational research in the future.

The advent of fast and cost-effective next-generation sequencing technologies have revolutionized the study of genomics and molecular biology in non-model plants that were previously deemed inaccessible. These high-throughput platforms enable us to study the unique structural organization of genes and the regulatory mechanisms underlying gene expression patterns, generate catalogs of specialized metabolism at the species level, and allow evolutionary analysis of genes, enzymes and pathways across species (Unamba *et al.*, 2015). The increased availability of genomic sequencing data has also shed light on the phenomenon of gene fusions in the biosynthesis of plant natural products. Hagel and Facchini (2017) comprehensively describe the gene fusions implicated in plant metabolism, discuss the possible mechanisms of their origin from an evolutionary perspective, explore the impact of fusion events in metabolism, and outline the potential uses of gene fusions in biotechnology (Hagel and Facchini, 2017).

The ever-growing technological advances in modern biotechnology today allow the precise engineering of new traits in plants within a short time, as opposed to traditional plant breeding. The CRISPR/Cas system allows the engineering of plants without residual exogenous DNA, which is beneficial for USDA approval of the final product. Advances in technologies and methodologies are revolutionizing plant engineering at several levels. Larsen *et al.* (2017) present interesting new advances in the functional characterization of transporters, proteins integral for cellular function. Using a combination of *in silico*, function- and phenotype-driven screens, several plant transporters have been identified and functionally characterized in different plant species (Larsen *et al.*, 2017). Although transport proteins are not primary engineering targets for trait improvement in plants, they offer innovative engineering applications for enhancing yield and plant performance under stress, both of which are interesting from an agricultural perspective. In fact, next-generation sequencing data have enriched our understanding of natural genetic diversity among plant membrane transporters, and allowed their engineering in crop plants like wheat and rice for sustainable food production even under severe environmental conditions (Schroeder *et al.*, 2013).

## Today’s companies

Besides trait engineering in plants with a view to augmenting human health, the production of high-value phytochemicals using synthetic biology approaches is a reality today. Companies like Evolva (evolva.com) and Amyris (amyris.com) take pride in using synthetic biology strategies for solving the supply-chain issue of nature through the bulk production of plant natural compounds in microbial organisms. There is an upsurge in biotech startup companies like Ginkgo Bioworks (ginkgobioworks.com) that are based on engineering microbes for customers across multiple markets. Through its foundries, this organism-engineering company has scaled and automated the process of making all sorts of chemicals in microorganisms. These companies primarily focus on metabolically engineering organisms to make profitable amounts of high-demand compounds that find applications in nutrition, health and personal care products, flavors and fragrances, and cosmetic additives. Moses *et al.* (2017) provide a systematic guide for the engineering of biorefineries from unicellular chassis using rational design principles, whilst emphasizing the choice of chassis as a key determinant for successful engineering (Moses *et al.*, 2017). It cannot be reiterated enough that synthetic biology has simplified and made achievable the design and construction of novel biological systems to screen for and produce new bioactives, food additives, fragrances, dietary supplements, and many other high-value chemicals.

## Future prospects

Plants are poised for a comeback as technological advances unravel unique properties and applications for diverse phytochemicals, either as pharmaceuticals or nutraceuticals to boost human health (Box 1). High-throughput sequencing technologies will generate a torrent of data in the coming decade from several ongoing multi-species plant projects. The affordability and ever-increasing sensitivity of these techniques has rendered sequencing of non-model, difficult to cultivate, and slow-growing plants plausible. Indexing species-specific phytochemical signatures will enable effective rational bioprospecting in the future. Alongside good cataloging of phytochemicals, it is also time to expand engineering toolkits for both plant biotechnology and synthetic biology to include less obvious molecular targets. For instance, the engineering of exporters in microorganisms would obviously facilitate the export of the product out of the cell, but in addition could also circumvent feedback inhibition and compound-associated toxicity, which in turn can increase yields and enable cost-effective downstream purification of the product.

**Box 1. Resurrecting the long-forgotten connection between plants and health**

**Figure:**
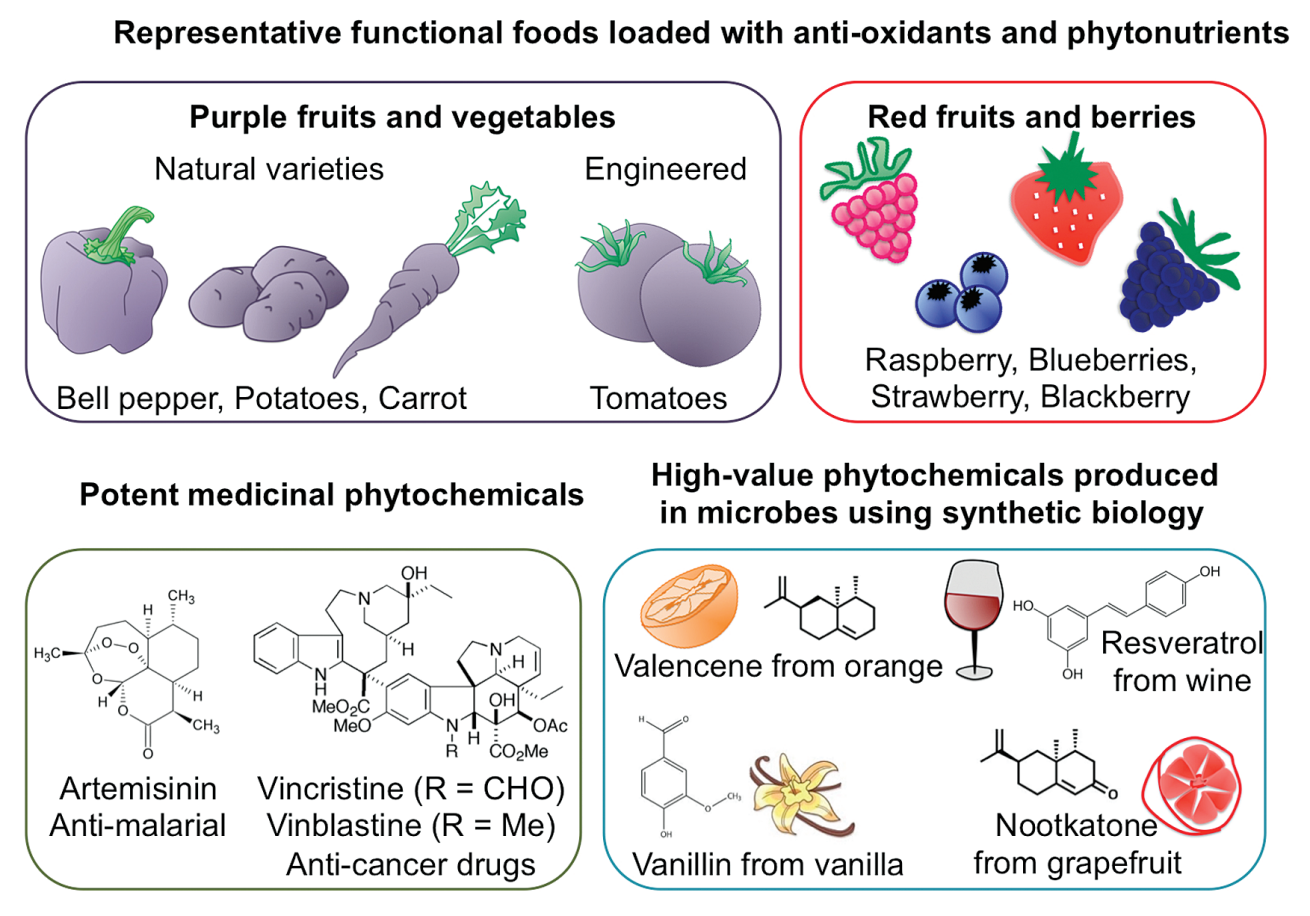

A big challenge still for phytochemical-based therapeutics is integrating the discovery of complex biosynthetic pathways with better characterization of molecular targets for the prevention and treatment of complex diseases. Likewise, assessing the compatibility of multifunctional phytochemicals and complex multicomponent plant extracts for disease-specific treatment is a challenge. However, contemplating the use of natural products in functional medicinal foods as a means of disease prevention, rather than treatment, is highly conceivable. As holistic approaches to prevention and treatment of sickness are increasingly gaining popularity, convincing the consumer market to make food choices for health benefits might be easier than currently anticipated. Plants easily represent the most abundant renewable source of high-value compounds, as they have evolved to synthesize a range of complex natural products efficiently. In addition, the upsurge in energy and chemical raw material costs, together with environmental concerns of carbon dioxide emission are detrimental to the pharmaceutical manufacturing of plant compounds. The result of dramatic advances in metabolic engineering, synthetic biology, genomics, proteomics, functional and molecular characterization, and pharmaceutical or nutraceutical screening, sets the path forward for botanical therapeutics. In the future, farmers who adapt their farming practices for the production of crops that promote health rather than provide calories are predicted to profit, and as a result the planet could become greener and healthier.

## References

[CIT0001] Hagel JM, Facchini PJ 2017 Tying the knot: occurrence and possible significance of gene fusions in plant metabolism and beyond. Journal of Experimental Botany 68, 4029–4043. 28521055 10.1093/jxb/erx152

[CIT0002] Larsen B, Xu D, Halkier BA, Nour-Eldin HH 2017 Advances in methods for identification and characterization of plant transporter function. Journal of Experimental Botany 68, 4045–4056. 28472492 10.1093/jxb/erx140

[CIT0003] Liu R, Li X, Lam KS 2017 Combinatorial chemistry in drug discovery. Current Opinion in Chemical Biology 38, 117–126. 28494316 10.1016/j.cbpa.2017.03.017 PMC5645069

[CIT0004] Martin C, Zhang Y, Tonelli C, Petroni K 2013 Plants, diet, and health. Annual Review of Plant Biology 64, 19–46. 10.1146/annurev-arplant-050312-120142 23451785

[CIT0005] Mendelsohn R, Balick MJ 1995 The value of undiscovered pharmaceuticals in tropical forests. Economic Botany 49, 223–228.

[CIT0006] Moses T, Mehrshahi P, Smith AG, Goossens A 2017 Synthetic biology approaches for the production of plant metabolites in unicellular organisms. Journal of Experimental Botany 68, 4057–4074. 10.1093/jxb/erx119 28449101

[CIT0007] Panche AN, Diwan AD, Chandra SR 2016 Flavonoids: an overview. Journal of Nutritional Science 5, 1–15. 10.1017/jns.2016.41 PMC5465813 28620474

[CIT0008] Pandeya SN, Thakkar D 2005 Combinatorial chemistry: A novel method in drug discovery and its application. Indian Journal of Chemistry 44, 335–348.

[CIT0009] Schroeder JI, Delhaize E, Frommer WB 2013 Using membrane transporters to improve crops for sustainable food production. Nature 497, 60–66. 23636397 10.1038/nature11909 PMC3954111

[CIT0010] Sliwoski G, Kothiwale S, Meiler J, Lowe EW Jr 2014 Computational methods in drug discovery. Pharmacological Reviews 66, 334–395. 24381236 10.1124/pr.112.007336 PMC3880464

[CIT0011] Sumner J 2000 The natural history of medicinal plants. Portland, OR: Timber Press.

[CIT0012] Tohge T, de Souza LP, Fernie AR 2017 Current understanding of the pathways of flavonoid biosynthesis in model and crop plants. Journal of Experimental Botany 68, 4013–4028. 10.1093/jxb/erx177 28922752

[CIT0013] Unamba CI, Nag A, Sharma RK 2015 Next generation sequencing technologies: the doorway to the unexplored genomics of non-model plants. Frontiers in Plant Science 6, 1074. 26734016 10.3389/fpls.2015.01074 PMC4679907

[CIT0014] Van Wyk BE 2015 A review of commercially important African medicinal plants. Journal of Ethnopharmacology 176, 118–134. 26498493 10.1016/j.jep.2015.10.031

